# Metallo-Curcumin-Conjugated DNA Complexes Induces Preferential Prostate Cancer Cells Cytotoxicity and Pause Growth of Bacterial Cells

**DOI:** 10.1038/s41598-018-33369-z

**Published:** 2018-10-08

**Authors:** Srivithya Vellampatti, Gopalakrishnan Chandrasekaran, Sekhar Babu Mitta, Vinoth-Kumar Lakshmanan, Sung Ha Park

**Affiliations:** 1 Sungkyunkwan Advanced Institute of Nanotechnology (SAINT) and Department of Physics, Sungkyunkwan University, Suwon, 16419 Korea; 2 Department of Biomedical Sciences, Chonnam National University Medical School, Gwangju, 61469 Korea; 3 Department of Biomedical Engineering, Sri Shakthi Institute of Engineering and Technology, Coimbatore, 641062 India

## Abstract

DNA nanotechnology can be used to create intricate DNA structures due to the ability to direct the molecular assembly of nanostructures through a bottom-up approach. Here, we propose nanocarriers composed of both synthetic and natural DNA for drug delivery. The topological, optical characteristics, and interaction studies of Cu^2+^/Ni^2+^/Zn^2+^-curcumin-conjugated DNA complexes were studied using atomic force microscopy (AFM), UV-vis spectroscopy, Fourier transform infrared and mass spectroscopy. The maximum release of metallo-curcumin conjugates from the DNA complexes, triggered by switching the pH, was found in an acidic medium. The bacterial growth curves of *E. coli* and *B. subtilis* displayed a prolonged lag phase when tested with the metallo-curcumin-conjugated DNA complexes. We also tested the *in vitro* cytotoxicity of the metallo-curcumin-conjugated DNA complexes to prostate cancer cells using an MTS assay, which indicated potent growth inhibition of the cells. Finally, we studied the cellular uptake of the complexes, revealing that DNA complexes with Cu^2+^/Ni^2+^-curcumin exhibited brighter fluorescence than those with Zn^2+^-curcumin.

## Introduction

Prostate cancer is a non-cutaneous male cancer, and along with lung cancer is the leading cause of cancer-related deaths in men. One in five men will be diagnosed with prostate cancer in their lifetime due to the genetic and biological mutations associated with aging. Conventional chemotherapy involves the use of anti-cancer drugs such as docetaxel and mitoxantrone, which affect not only cancer cells but also neighbouring healthy cells (*e.g*., bone marrow, where new blood cells are synthesized). In 2017, prostate cancer was estimated to account for 21, 9.9, and 9.6% of all new cancer cases in Canada, Korea, and the USA, respectively. In the last 20 years, research has been conducted to address the issues related to chemotherapy so that cancer can be treated promptly. To this end, the nanotechnology has been utilized in multiple medical disciplines such as therapies and diagnostics^1–3^, biosensors^4^, and optoelectronics^5–7^, to advance the prevention and treatment of cancer.

DNA nanotechnology, a sub-field of nanotechnology, involves programming and designing nanostructures from DNA molecules rather than treating them as carriers of hereditary information. The diverse potential structures of DNA molecules due to their inherent base-sequence programmability and self-assembly by hydrogen bonding between bases has resulted in DNA being widely recognized as one of the most prominent nanoengineering materials^8–10^. This technology is a fusion of various disciplines such as biology, physics, chemistry, and electronics, allowing us to use it in biomedicine and create a variety of functionalized devices and sensors^11–18^. Consequently, researchers are focusing on the construction of diverse shapes of DNA nanostructures for *in vitro* and *in vivo* medical applications (including DNA vehicles for drug delivery) as well as for conventional electromagnetic and optical devices and chemical and biological sensors. Some examples of DNA nanostructures used in drug delivery are DNA origami, tetrahedra, icosahedra, nanorobots, and nanocages^12,19–24^.

**Figure 1:**
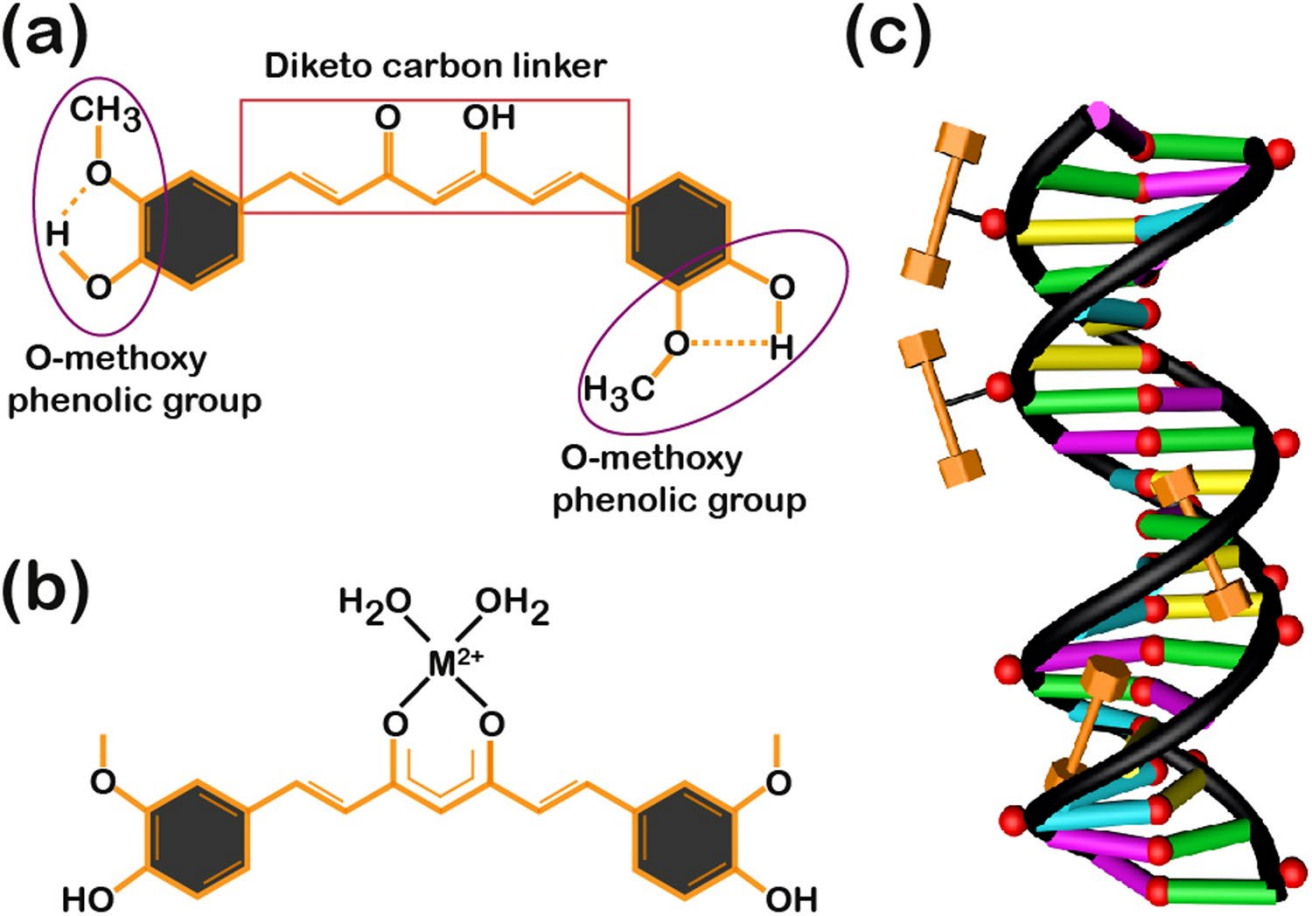
Schematic representations of curcumin, metallo-curcumin, and metallo-curcumin binding to DNA. (**a**) The molecular structure of curcumin, with functional groups containing two methoxy-phenolic moieties connected by a seven-carbon linker. (**b**) Structure of metallo-curcumin, showing the deprotonation of the hydroxyl group in the keto-enol tautomeric group which forms a bidentate di-ketone group where the ion binds. M^2+^ represents one of the divalent metal ions, namely Cu^2+^, Ni^2+^, and Zn^2+^. (**c**) Schematic representation of metallo-curcumin binding to the minor groove and the phosphate backbone of the DNA duplex. The orange dumbbell-shaped structures represent curcumin molecules and the red spheres in the curcumins represent M^2+^.

Curcumin (di-feruloylmethane) is a biologically active component of the Indian spice known as turmeric (Curcumin longa), which belongs to the Zingiberaceae family. Turmeric is used as a conventional medicine to treat joint inflammation, cold, flu, stomach and liver problems, viral and bacterial infections, some neurodegenerative diseases including Parkinson’s and Alzheimer’s, and cancer because of its anti-oxidative and anti-cancer properties^25,26^. It induces apoptosis in various carcinomas, especially prostate cancers, by down-regulating TNF-inducible NF-κB and AP-1 protein activity. Curcumin has a molar mass of 368.38 g/mol and is mildly soluble in water but quite soluble in dimethyl sulfoxide, acetone, and ethanol. Curcumin is a monobasic bi-dentate molecule with three functional groups: a phenolic moiety, a keto-enol tautomeric group, and a seven-carbon linker molecule (Fig. 1(a)). The aromatic group induces π-π interactions, whereas the keto-enol and phenolic groups are involved in hydrogen bonding with the target macromolecules. The phenolic group is responsible for the anti-oxidant activity of curcumin. The carbon linker molecule provides the flexibility to adapt to specific biomolecule conformations and increase hydrophobic interactions with the protein^27,28^. Despite its characteristic pharmacodynamic behaviour (chemopreventive, cytotoxic, and anti-metastatic properties), curcumin is pharmacokinetically feeble (for example, its instability reduces bioavailability and systemic uptake), which is a severe drawback^29^. Over the last few years, some strategies have been successful in taking advantage of curcumin’s therapeutic behaviours, such as encapsulating it in cyclodextrins and conjugating it with silver nanoparticles^30–32^. In one of these strategies, researchers reported chelating curcumins with metal ions and vanadyl-indium complexes to enhance their pharmacodynamic effects and diminish cytotoxic potential^33–40^. Keto-enol (β-di-ketone moiety)—a group in which the metal ion is bound by covalent interactions—induces structural variation as shown in Fig. 1(b). Here, a divalent metal ion (M^2+^) improves the solubility and therapeutic efficacy of the curcumin^41^. Although curcumin mainly binds to DNA *via* groove binding, a metallo-curcumin conjugate enables additional bonds with DNA through electrostatic interaction and intercalation^42^.

Here, we developed metal-ion-chelated curcumin conjugated with DNA nanostructures to serve as drug carriers (constructed with salmon DNA (SDNA) and DNA rings (RDNA) by sequence-designed synthetic oligomers) and examined their anti-bacterial and anti-cancer activity (Fig. 1(c)). We chose three metal ions, namely Cu^2+^, Ni^2+^, and Zn^2+^, for chelating the curcumin and analysed the interactions of the metallo-curcumin conjugates with DNA using physical characterization methods such as UV-vis, Fourier transform infrared (FTIR) and mass spectroscopies. The *in vitro* release of metallo-curcumin from the RDNA complexes was examined under two different pH conditions to validate the delivery of the drug (*i.e*., metallo-curcumin) at the molecular level by bypassing several physiological barriers. Furthermore, growth inhibition studies of bacteria (*E. coli* and *B. subtilis*) and the cell viability profiles of five different prostate cancer cells, namely PC3 (human prostate cancer cells derived from bone metastasis), LNCaP (human metastatic prostate carcinoma found in lymph nodes), TRAMP-C1 (transgenic adenocarcinoma mouse prostate model), 22Rv1 (human prostate carcinoma isolated from bone metastasis), and DU145 (human prostate carcinoma isolated from brain metastasis), were used to determine the anti-bacterial and anti-cancer activity of the metallo-curcumin-conjugated DNA complexes. Finally, fluorescence microscopy was used to visualize the cellular internalization of metallo-curcumin-conjugated RDNA complexes in 22Rv1 cells, showing a substantial uptake of curcumin into the cells.

## Results

### Curcumin and metallo-curcumin binding to DNA

Typically, third-party molecules bind to DNA in three different ways: electrostatic interaction, intercalation, and groove binding. Curcumin (abbreviated hereafter as C) binds mostly to DNA *via* groove binding^43^. Groove binding is related to the lock-and-key model for drug binding to DNA (since the drugs adjust their structures to follow the groove as the DNA twists around its central axis, as shown in Fig. 1(c), which has been proven clinically as an anti-bacterial and anti-cancer agent^25^. We initially checked the structural stability of DNA structures (DNA rings without deformation and SDNA without precipitation) with the addition of a certain concentration of metallo-curcumin conjugates (to achieve significant function enhancement). We chose 4 mM of Cu^2+^, 2 mM of Ni^2+^, and 1 mM of Zn^2+^ with 80 µM of C at a fixed concentration of either RDNA (500 nM) or SDNA (0.1 wt%) because these concentrations were sufficient to reveal the specific function of the metallo-curcumin conjugate (anti-bacterial and anti-cancer activity) without disturbing the DNA structure, which is discussed below^44^. The RDNA (SDNA) complexes with Cu^2+^ and C, Ni^2+^ and C, and Zn^2+^ and C conjugates were named RDNA-Cu-C, RDNA-Ni-C, and RDNA-Zn-C (SDNA-Cu-C, SDNA-Ni-C, and SDNA-Zn-C), respectively.

### Structural morphology of curcumin- and metallo-curcumin-conjugated RDNA complexes

**Figure 2:**
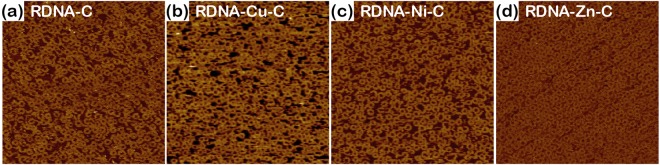
AFM images of curcumin-conjugated and metallo-curcumin-conjugated RDNA complexes. The outer and inner diameters of the RDNA were 29 and 13 nm, respectively. (**a**) AFM image of C-conjugated RDNA complexes, labelled RDNA-C. (**b–d**) AFM images of Cu^2+^-, Ni^2+^-, and Zn^2+^-curcumin-conjugated RDNA complexes, labelled RDNA-Cu-C, RDNA-Ni-C, and RDNA-Zn-C, respectively. The final concentrations of RDNA, C, Cu^2+^, Ni^2+^, and Zn^2+^ were 500 nM, 80 µM, 4 mM, 2 mM, and 1 mM, respectively. The scan size of all the images was 2 × 2 µm^2^.

AFM analysis was performed for the RDNA to verify its structural stability after doping with metallo-curcumin conjugates with [C] of 80 µM, [Cu^2+^] of 4 mM, [Ni^2+^] of 2 mM, and [Zn^2+^] of 1 mM. We observed that the RDNAs doped with metallo-curcumin conjugates were well assembled with the expected geometry (Fig. 2). The RDNA formed a ring structure with an outer diameter of ~29 nm by hybridization of the two strands^45,46^.

### Spectroscopic evidence of curcumin- and metallo-curcumin, metallo-curcumin-conjugated SDNA complexes

**Figure 3:**
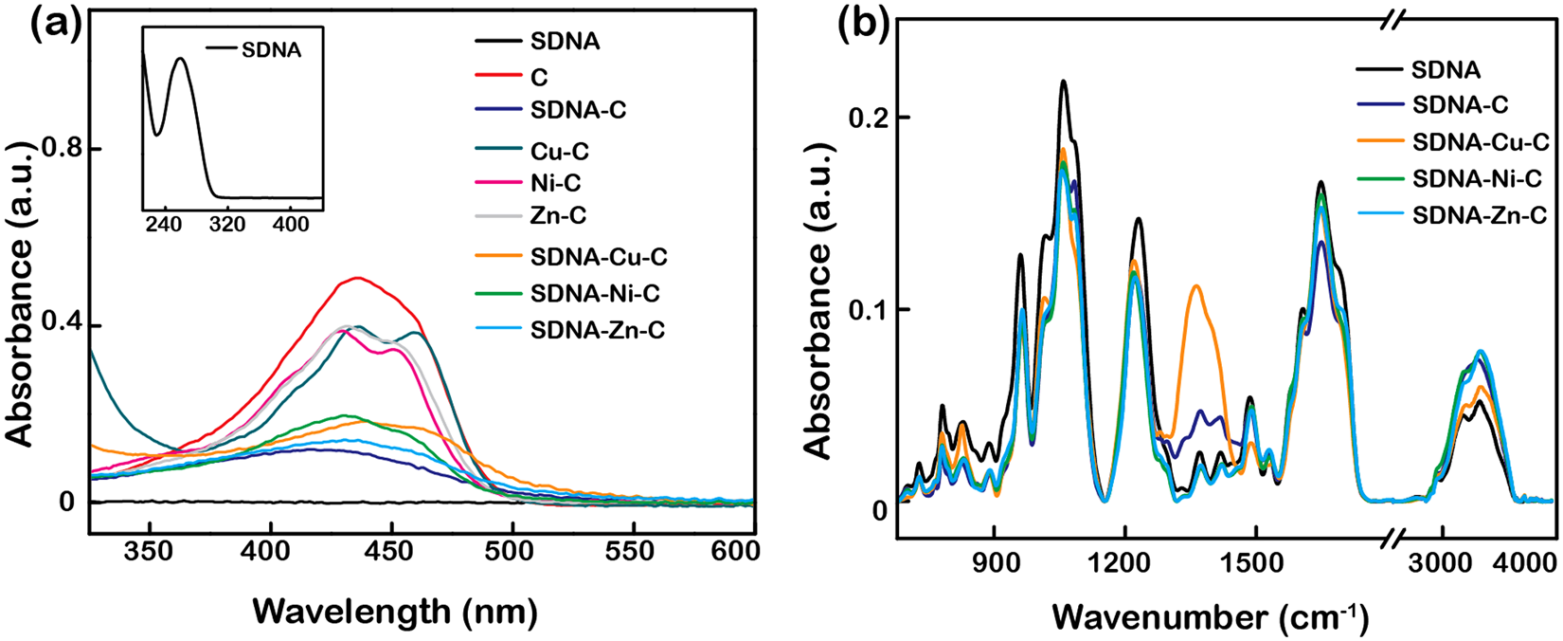
Spectroscopic measurements of SDNA, curcumin, metallo-curcumin, curcumin-conjugated SDNA complexes, and metallo-curcumin-conjugated SDNA complexes. (**a**) UV-vis absorption spectra of SDNA, C, C with Cu^2+^, C with Ni^2+^, C with Zn^2+^, curcumin-conjugated SDNA, and Cu^2+^-, Ni^2+^-, and Zn^2+^-curcumin-conjugated SDNA complexes. Inset shows the characteristic peak of DNA at 260 nm. (**b**) FTIR absorption spectra of SDNA, SDNA-C, SDNA-Cu-C, SDNA-Ni-C, and SDNA-Zn-C. Final concentrations of SDNA, C, Cu^2+^, Ni^2+^, and Zn^2+^ were 0.1 wt%, 80 µM, 4 mM, 2 mM, and 1 mM, respectively.

The photo physical behaviour of the metallo-curcumin conjugates and their interactions with the SDNA were investigated using UV-vis and infra-red spectroscopy, as shown in Fig. 3. Figure 3(a) shows a UV-vis spectrum with an intense band for C at 435 nm due to π-π interactions. In the case of the metallo-curcumin-conjugated SDNA complexes, we observed absorption shifts of 1–6 nm and shoulder peaks at around 450 nm. The shoulder peaks contributed to the metal complexation with C and variation in the shoulder peaks depended on the type of metal ion^47,48^. To further study the physical interactions within the metallo-curcumin-conjugated SDNA complexes, we performed a qualitative analysis of the interactions between the DNA and the metallo-curcumin conjugates.

FTIR spectroscopy was performed to qualitatively investigate the interactions of the metallo-curcumin-conjugated SDNA complexes, as displayed in Fig. 3(b). The DNA signature peaks were divided into five regimes; their assignments are tabulated in detail in Supplementary Table S3. 3650–3000 cm^−1^ corresponded to ‒OH stretching, 1800–1500 cm^−1^ to in-plane base vibrations, 1500–1250 cm^−1^ to base-sugar vibrations, 1250–1000 cm^−1^ to sugar-phosphate vibrations, and finally 1000–700 cm^−1^ to sugar vibrations. Interestingly, the peak at 3330 cm^−1^ (the band attributed to the phenolic group) did not change compared to that of bare C even after chelating metal ions. This indicates that the phenolic ‒OH group was not involved in the metal complexation process. The peak intensities at 1016, 1058, and 1083 cm^−1^ in the phosphate band region (1250–1000 cm^−1^) were lower than those of pristine SDNA, which may be related to the specific binding of the metallo-curcumin conjugates to the DNA helix^48,49^. The SDNA peak at 1230 cm^−1^ (the PO_2_^−^ antisymmetric stretching mode) shifted to a lower frequency of ~1224 cm^−1^ and decreased in intensity upon metallo-curcumin complexation. The *B*-form markers at 830 cm^−1^ for the metallo-curcumin-conjugated SDNA complexes indicated the presence of the *B*-form of DNA with little disturbance. With the help of the binding and interaction patterns obtained by UV-vis and FTIR measurements, we will test the drug release profile of the metallo-curcumin-conjugated DNA complexes.

**Figure 4:**
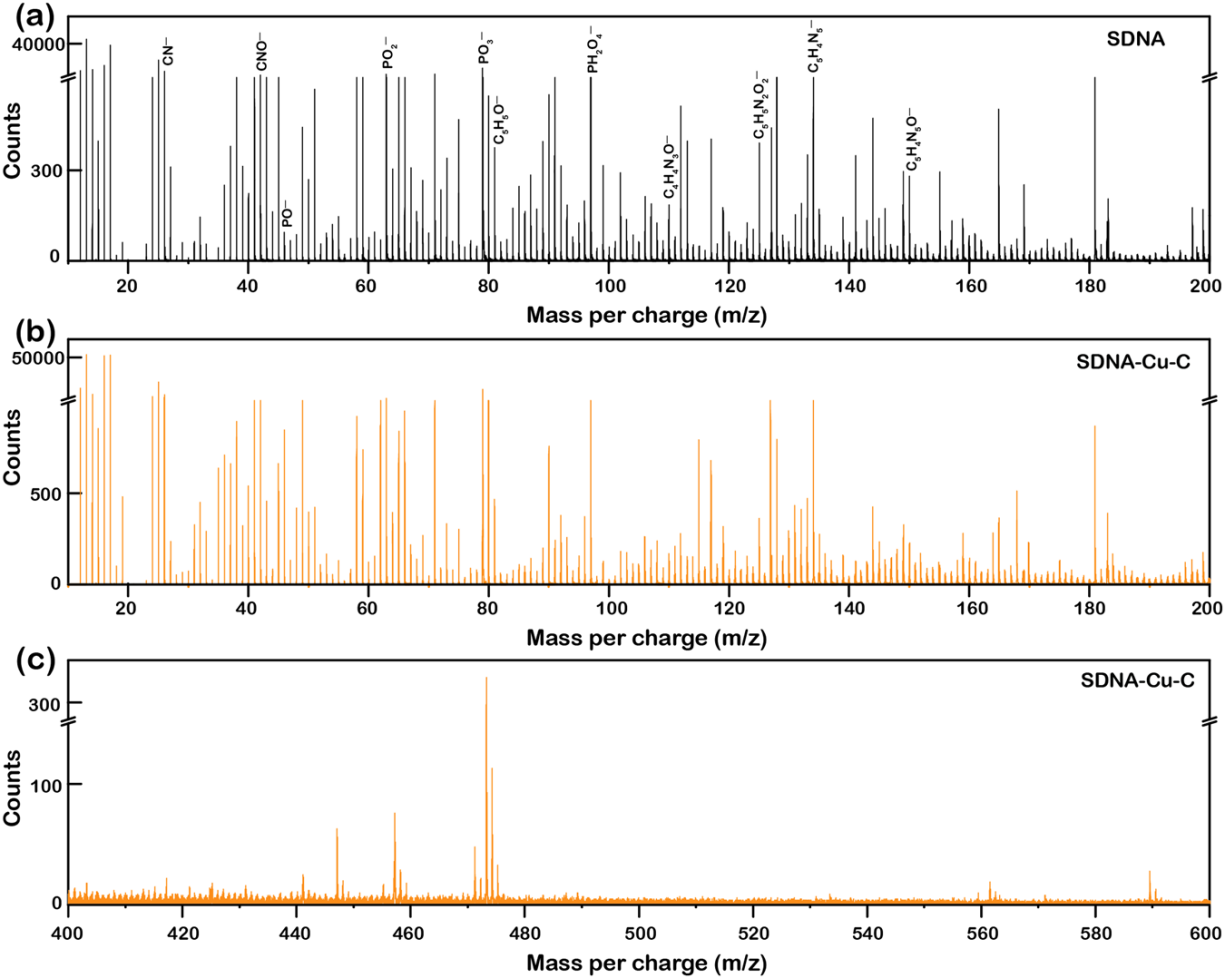
Representative negative ion mode TOF-SIMS spectra of the pristine SDNA and SDNA-Cu-C complexes. (**a**) TOF-SIMS spectrum of pristine SDNA molecules showing the various chemical fragments produced from sugar, phosphate backbone, and nucleobase with corresponding mass-to-charge ratios (*m/z*) in the range from 10 to 200 as indicated in the graph. (**b,c**) TOF-SIMS spectra of the SDNA-Cu-C complex revealing DNA and Cu-C chemical fragments in the ranges of *m/z* of 10‒200 and 400‒600, respectively.

For the detection and distribution of the molecular species exist in DNA and SDNA-Cu-C complexes, we performed the time-of-flight secondary ion mass spectroscopy (TOF-SIMS) (Fig. 4). DNA molecules were composed of deoxyribose sugar, phosphate groups, and nucleobases, which were detected as DNA molecular ion fragments using the negative ion mode TOF-SIMS. Figure 4(a) displayed the negative ion mode TOF-SIMS spectra of the SDNA molecules with corresponding mass-to-charge ratios (m/z) in the range from 10 to 200. The chemical fragment peaks of CN^−^, CNO^−^, and C_5_H_5_O^−^ at *m/z* of 26, 42, and 81, respectively indicate the presence of deoxyribose sugars. The chemical fragment peaks of PO^−^, PO_2_^−^, PO_3_^−^, and PH_2_O_4_^−^ at *m/z* of 47, 63, 79, and 97, respectively confirm the existence of phosphate groups. The peaks of C_4_H_4_N_3_O^−^ (cytosine), C_5_H_5_N_2_O_2_^−^ (thymine), C_5_H_4_N_5_^−^ (adenine), and C_5_H_4_N_5_O^−^ (guanine), which correspond to *m/z* of 110, 125, 134, and 150, respectively, verify the specific nucleobases^50–52^.

Figures 4(b,c) illustrated the negative ion mode TOF-SIMS spectra of the SDNA-Cu-C complexes in the ranges of *m/z* of 10‒200 and 400‒600, respectively. As we expected, the characteristic chemical fragments of DNA in the SDNA-Cu-C complex showed similar *m/z* values as compared to the pristine SDNA. In addition, the chemical fragment of Cu-C in SDNA was revealed at the corresponding *m/z* of 431. Interestingly, the chemical fragments *i.e*., C_5_H_4_N_5_-Cu-C_5_H_5_N_2_O_2_ (between adenine and thymine) and C_5_H_4_N_5_O-Cu-C_4_H_4_N_3_O (between guanine and cytosine), which were produced by the interaction between the ions and nucleobases, exhibited weak peak signals at *m/z* of 322 and 323, respectively due to the opposite charge characteristics between ions and DNA molecules. The chemical distribution of the individual chemical fragments of DNA and SDNA-Cu-C were obtained by the negative ion mode SIMS mapping. The mapping images of characteristic molecular ion species (CN^−^, CNO^−^, PO_2_^−^, PO_3_^−^, PH_2_O_4_^−^, C_4_H_4_N_3_O^−^, C_5_H_4_N_5_O^−^, C_5_H_5_N_2_O_2_^−^, and C_5_H_4_N_5_^−^) in both SDNA and SDNA-Cu-C were shown in the Supplementary Fig. S2. From the observation of images, the chemical fragments in both pristine SDNA and SDNA-Cu-C complexes were homogenously distributed.

### *In vitro* drug release kinetics

**Figure 5:**
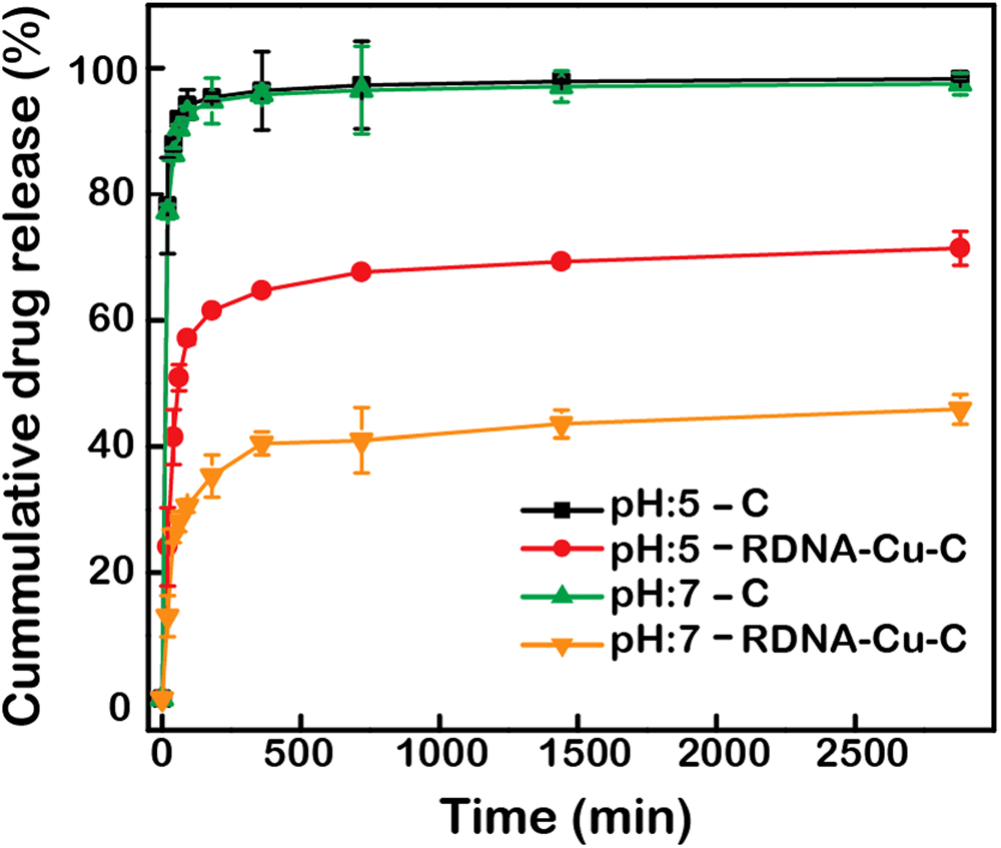
*In vitro* release of curcumin and metallo-curcumin conjugates from RDNA under two different pH conditions. The release rate of C from the Cu^2+^-curcumin-conjugated RDNA complexes was analysed using 0.2 M phosphate buffered saline (pH 5 and pH 7) containing 0.5% Tween 20 for two days. The data represent the mean values of three measurements with error bars.

The cumulative Cu-C release profile from the RDNA nanostructures was measured at pH 7 and 5 at 37 °C for 2 days, as shown in Fig. 5. The free C profile was treated as a control. Cu-C (here, M^2+^ enhances the solubility and therapeutic efficacy of C) is specifically discussed because similar patterns were observed for the release profiles of the other metallo-curcumin conjugates^53^. Free C displayed a similar pattern of burst release at both pH 7 and 5 (no noticeable differences with respect to pH), with ~95.8% and ~96.4% measured at 6 h, respectively. In contrast, the release of Cu-C from RDNA-Cu-C complexes was clearly different between pH 7 and pH 5. Two distinct phases (initial burst release and slower release regions) were observed. Around 64.7% of Cu-C was released from the RDNA at pH 5 after 6 h, whereas only 40.5% was released at pH 7. Finally, Cu-C release reached around 71.4% and 45.86% for pH 5 and 7, respectively. While DNA is stable in physiological conditions (*e.g*., blood pH of 7), pH-responsive DNA nanocarriers release their maximum payloads in acidic surroundings (*e.g*., lysosomal pH of 5) like an effective drug delivery system. Based on our results, metallo-curcumin-conjugated RDNA complexes also showed maximum release in acidic pH due to the disintegration of the phosphodiester bonds in the DNA structure, which releases the drug^24^. Sustained release of drugs helps improve drug efficacy, patient compliance, and toxicity, motivating the development of new nanomaterials.

### Anti-bacterial assay of metallo-curcumin-conjugated DNA complexes against *E. coli* and *B. subtilis*

**Figure 6:**
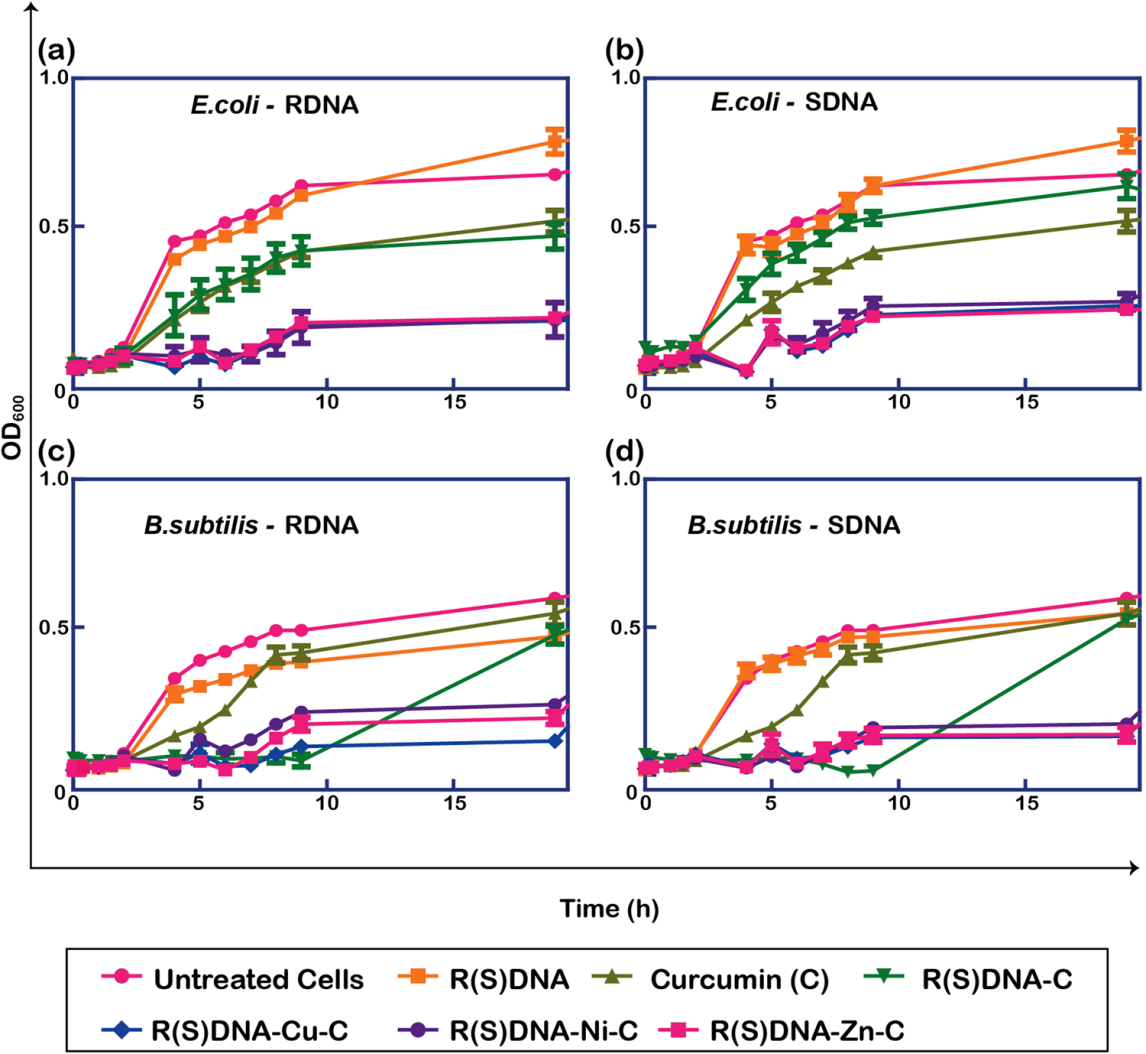
Representative bacterial population growth inhibition curves for *E. coli* and *B. subtilis* upon treatment with RDNA or SDNA, curcumin, curcumin-conjugated R(S)DNA, metallo-curcumin-conjugated R(S)DNA complexes in LB medium for 20 h. (**a,b**) Growth rate measured by optical density at 600 nm (OD_600_) of *E. coli* with and without R(S)DNA, C, R(S)DNA-C, R(S)DNA-Cu-C, R(S)DNA-Ni-C, and R(S)DNA-Zn-C complexes at 37 °C. (**c,d**) Growth inhibition curves of *B. subtilis* with and without R(S)DNA, C, R(S)DNA-C, R(S)DNA-Cu-C, R(S)DNA-Ni-C, and R(S)DNA-Zn-C complexes at 37 °C. The curves show the mean of triplicate measurements, and the error bars represent the standard deviation of the mean. Untreated cells were used as a control.

To confirm the penetration and drug delivery of the metallo-curcumin-conjugated DNA complexes, we investigated the effects of the complexes in bacterial cells. Representative bacterial population growth inhibition curves of *E. coli* and *B. subtilis* upon treatment with metallo-curcumin-conjugated RDNA and SDNA complexes are shown in Fig. 6. Examining the bacterial growth kinetics, the growth of *E. coli* continuously increased in the control samples (untreated RDNA and SDNA) after a lag phase of 2.5–3 h. Free C and DNA-C (*i.e*., RDNA-C and SDNA-C) had a lag phase of 0.5 h and a mild reduction in growth rate compared to the controls. On the other hand, the metallo-curcumin-conjugated DNA complexes had a strong-delayed lag phase of 5.5 h, and the total growth of *E. coli* was arrested at around 8.5–9 h with ~60% population reduction compared to the controls, as shown in Fig. 6(a,b). The Gram-positive bacterium *B. subtilis* showed a similar trend to *E. coli*, in which the lag phase was delayed by about 6 h for the metallo-curcumin-conjugated DNA complexes. The growth of the bacterium was completely arrested at ~9 h with ~50% reduction in the population, as depicted in Fig. 6(c,d). However, the *B. subtilis* treated with either RDNA-C or SDNA-C showed an extended lag phase until 9 h followed by a sudden log phase. This implies that free C was highly efficient at inducing a prolonged bacteriostatic effect on *B. subtilis*. Consequently, the metallo-curcumin-conjugated DNA complexes had promising bacteriostatic and bactericidal effects on both the Gram-negative and Gram-positive bacterial strains. Finally, we aimed to test the effects of the metallo-curcumin-conjugated DNA complexes in mammalian prostate cancer cells to elucidate their anti-cancer properties.

### Cytotoxicity activity in human prostate cancer cell lines

**Figure 7:**
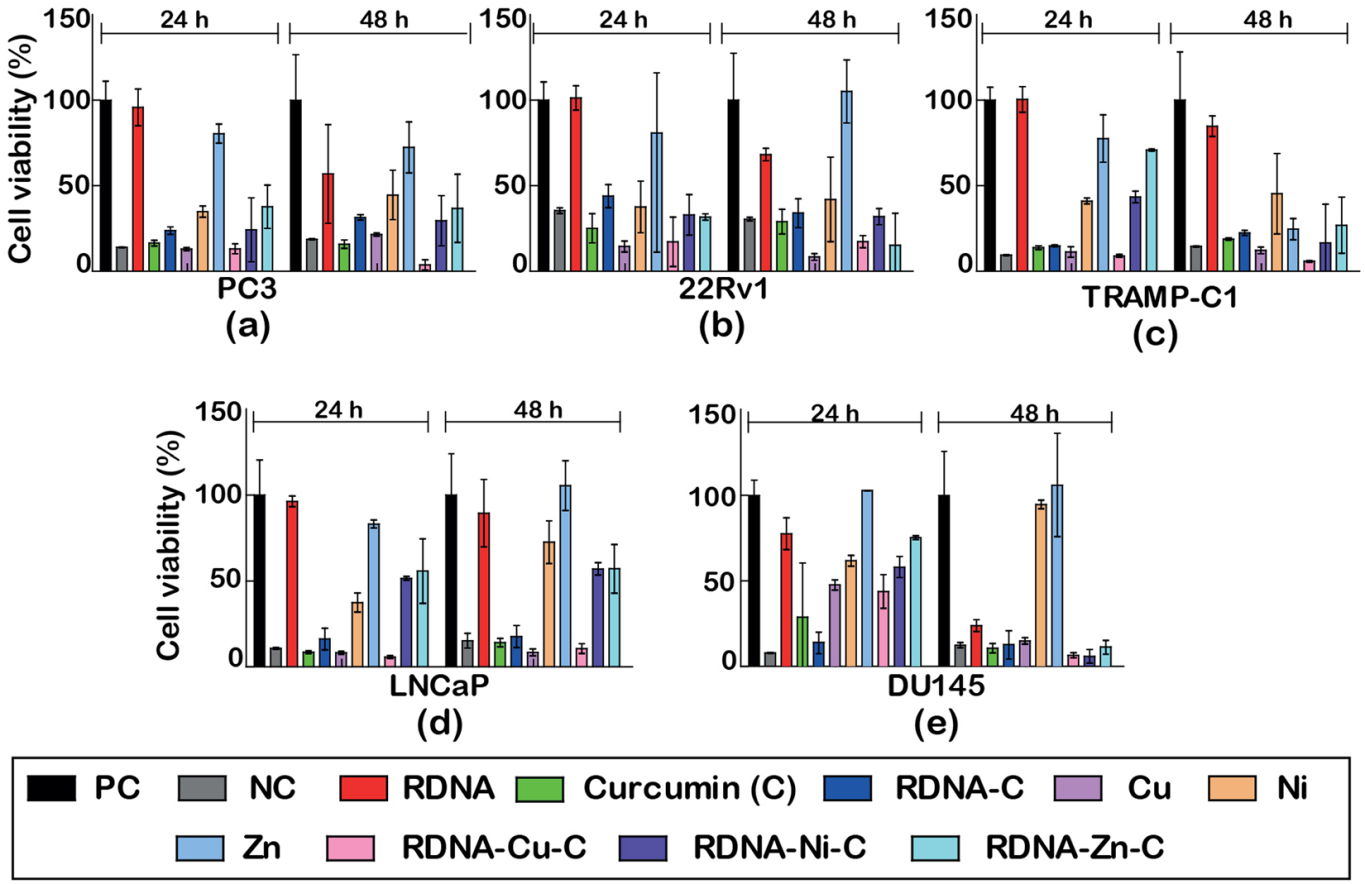
(**a–e**) Cell viability profiles of five different prostate cancer cell lines (PC3, 22Rv1, TRAMP-C1, LNCaP, and DU145) treated with RDNA, C, RDNA-C, Cu^2+^, Ni^2+^, Zn^2+^, RDNA-Cu-C, RDNA-Ni-C, and RDNA-Zn-C by MTS assay for 24 and 48 h. Untreated cells were used as a positive control and Triton X-100–treated cells as a negative control.

**Figure 8:**
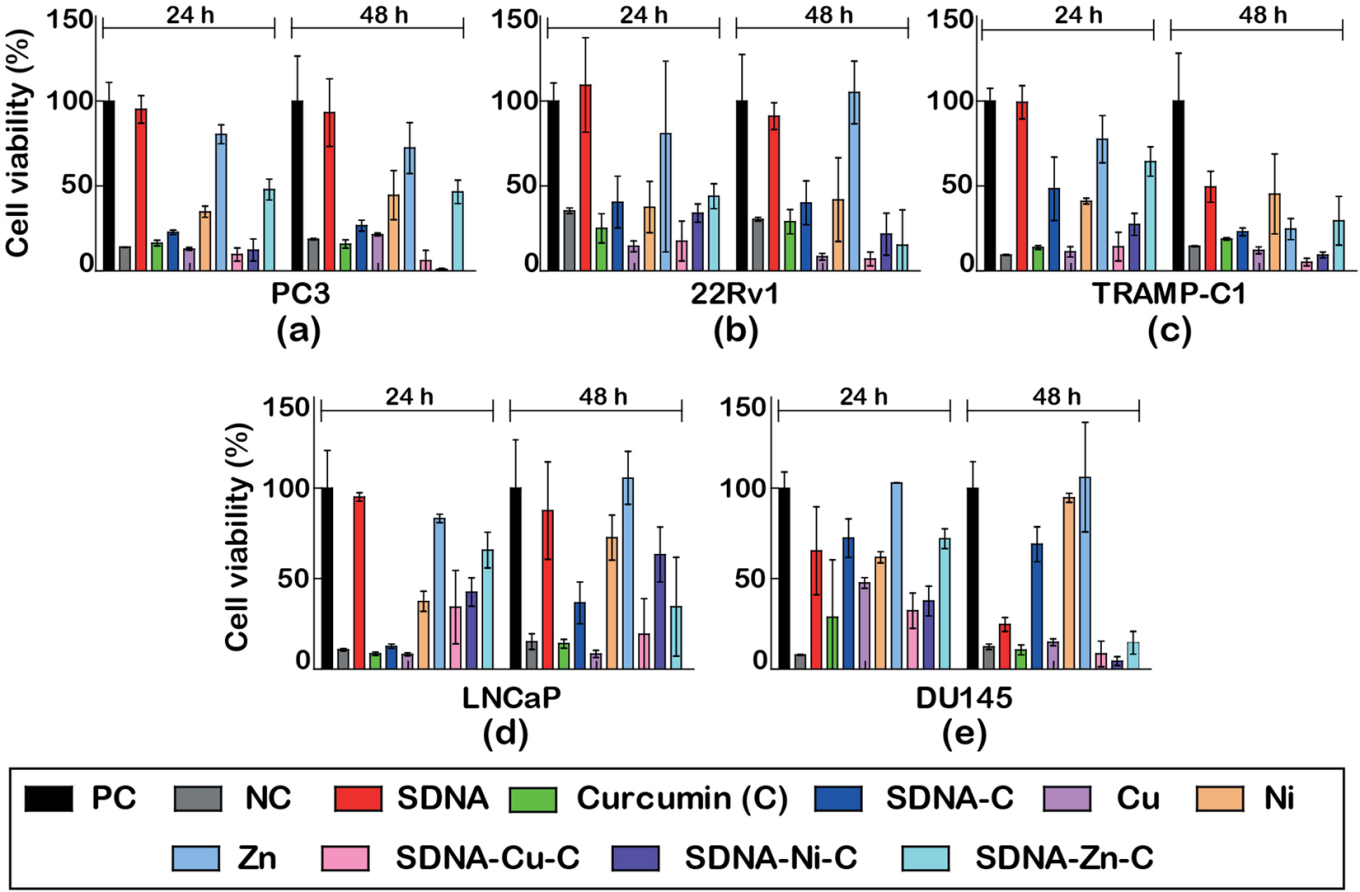
(**a–e**) Cell viability profiles of prostate cancer cell lines (PC3, 22Rv1, TRAMP-C1, LNCaP, and DU145) treated with SDNA, C, SDNA-C, Cu^2+^, Ni^2+^, Zn^2+^, SDNA-Cu-C, SDNA-Ni-C, and SDNA-Zn-C by MTS assay for 24 and 48 h. Untreated cells were used as a positive control and Triton X-100 treated cells as a negative control.

Figures 7 and 8 show the cell viability profiles of the metallo-curcumin-conjugated RDNA and SDNA complexes, respectively, on five different prostate cancer cell lines (PC3, 22Rv1, TRAMP-C1, LNCaP, and DU145). Equivalent concentrations of C, RDNA-C (SDNA-C), RDNA-Cu-C, RDNA-Ni-C, and RDNA-Zn-C (SDNA-Cu-C, SDNA-Ni-C, and SDNA-Zn-C) complexes were assessed by MTS colorimetric assay for 24 and 48 h. The metallo-curcumin-conjugated DNA (both RDNA and SDNA) complexes had a positive impact on both androgen-sensitive (LNCaP, 22Rv1, and TRAMP-C1) and androgen-independent (PC3 and DU145) prostate cancer cells. Interestingly, the cytotoxicity of the metallo-curcumin-conjugated DNA complexes was higher in DU145 cells (derived from brain metastasis) at 48 h than in the other cell lines. Based on the data, the order of cytotoxicity of the metallo-curcumin-conjugated DNA complexes in the cell lines was DU145 > PC3 > TRAMP-C1 > 22Rv1 > LNCaP.

After treating with DNA-C with different M^2+^, the cells showed different characteristic toxicities. RDNA-Cu-C complexes showed better cytotoxicity in the five different prostate cancer cells (83–96%) than RDNA-Ni-C (49–84%) and RDNA-Zn-C (30–74%) complexes (Fig. 7). Similarly, the cytotoxicity in cancer cells was significantly greater upon treatment with the SDNA-Cu-C complexes (83–95%) than with the SDNA-Ni-C (58–79%) or SDNA-Zn-C (35–71%) complexes (Fig. 8). The decreased cytotoxicity of the DNA-Zn-C conjugates was due to the presence of strong O‒H bonds in the O-methoxy phenolic group, which led to diminished scavenging activity compared to the other complexes which lose H-atoms more readily^39^. The synergistic effect of M^2+^ and C binding to DNA is possibly made more effective in cancer cells by inhibiting NF-κB and AP-1 activity, thereby activating apoptotic genes. The cytotoxicity of the cells treated with bare M^2+^, C, and DNA-C was lower than that of the cells treated with metallo-curcumin-conjugated DNA complexes.

### Cellular internalization of metallo-curcumin-conjugated RDNA complexes

**Figure 9:**
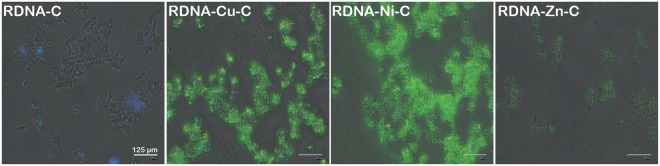
*In vitro* cellular uptake of RDNA-C, RDNA-Cu-C, RDNA-Ni-C, and RDNA-Zn-C complexes in 22Rv1 cells. The merged confocal images of 22Rv1 cells are visualized for curcumin fluorescence (in green). The metallo-curcumin-conjugated RDNA complexes were found to be well internalized by the cells to enable termination. The scale bars in all the images are 125 µm.

To validate the cellular uptake of the metallo-curcumin-conjugated RDNA complexes, we measured the *in vitro* cellular internalization in 22Rv1 cells treated with the complexes, as shown in Fig. 9. The complexes were internalized inside the cell through extrinsic pathways such as receptor-mediated endocytosis with vesicles known as early endosomes. These early endosomes mature and fuse with cytoplasmic vesicles to form late endosomes and lysosomes before activating apoptotic proteins, which subsequently leads to death^54^. RDNA-C was used as a comparison for the extent of uptake. C possesses a characteristic green fluorescence which was utilized to visualize the cellular uptake in the cells. Interestingly, RDNA-Cu-C and RDNA-Ni-C exhibited brighter fluorescence than RDNA-Zn-C. The lower uptake of RDNA-Zn-C coincided with the weak cytotoxicity observed in the MTS assay.

## Discussion

To summarize, we constructed Cu^2+^/Ni^2+^/Zn^2+^-curcumin-conjugated ring DNA and salmon DNA complexes. We characterized their topology using AFM and their optical properties and interaction studies using UV-vis, FTIR, and mass spectroscopy. Metal ions improved the solubility of the curcumin and provided additional DNA binding ability. In addition, we evaluated the anti-bacterial and anti-cancer activity of the complexes in terms of growth inhibition, cell viability, and cellular uptake through the *in vitro* pH-controlled release of metallo-curcumin from the DNA complexes. RDNAs doped with metallo-curcumin conjugates were well assembled with the expected geometry. The chemical binding of the metallo-curcumin conjugates to DNA and the characteristic peaks of the DNA were studied using UV-vis, FTIR and mass spectra. Based on release profiles, the metallo-curcumin-conjugated DNA complexes released a maximum quantity of conjugates in acidic pH due to the breaking of the phosphodiester bonds in the DNA structure. The metallo-curcumin-conjugated DNA complexes exhibited a strong-delayed lag phase in both Gram-positive and Gram-negative bacteria. Metallo-curcumin-conjugated DNA complexes showed significant toxicity to prostate cancer cells compared to pristine DNA. Finally, we performed cellular uptake studies which revealed that RDNA-Cu-C and RDNA-Ni-C internalized more efficiently than RDNA-Zn-C, as measured by bright fluorescence. Our results prove that DNA nanostructures without any transfection agents can improve drug loading, drug release, and anti-bacterial and anti-cancer activity, warranting their use as novel and efficient nanocarriers for drug delivery applications.

## Methods

### Synthesis of DNA rings

Equimolar concentrations of two different DNA strands (Ring 1–1 and Ring 1–2) were added to 1 × TAE/Mg^2+^ buffer (40 mM Tris(hydroxymethyl)aminomethane), 20 mM acetic acid, and 1 mM EDTA with 12.5 mM magnesium acetate). The final concentration of RDNA was 500 nM. The assembly of DNA rings in the test tube involved cooling from 95 °C to room temperature for 24 h to aid hybridization by placing the test tube in a Styrofoam box containing 2 L of boiling water. The annealed structures were incubated at 4 °C to stabilize the ring structure (Supplementary Fig. S1, Supplementary Tables S1 and S2.

### Preparation of salmon DNA

0.01 g of salmon DNA fibres were dissolved in 10 mL of deionized (DI) water and stirred overnight to obtain a homogeneous DNA solution. The final concentration of SDNA ([SDNA]) was 0.1 wt%.

### Synthesis of metallo-curcumin-conjugated DNA complexes

Metallo-curcumin conjugates were prepared by mixing the appropriate amount of metal ions to the curcumin solution in 10% DMSO. The metallo-curcumin conjugates in 10% DMSO were stirred for 4 h at room temperature. After preparing the conjugates, an appropriate amount was added to the DNA solution (either RDNA or SDNA) and left undisturbed for 24 h to incubate. Final [curcumin], [Cu^2+^], [Ni^2+^], and [Zn^2+^] in the conjugates were 80 µM, 4 mM, 2 mM, and 1 mM, respectively. Final [curcumin], [Cu^2+^], [Ni^2+^], [Zn^2+^], [RDNA], and [SDNA] in the metallo-curcumin-conjugated DNA complexes were 80 µM, 4 mM, 2 mM, 1 mM, 500 nM, and 0.1 wt%, respectively (Fig. 1).

### AFM imaging

A DNA sample solution was dropped on a mica substrate and left undisturbed for 5 min, followed by the addition of 20 µL of 1 × TAE/Mg^2+^ buffer. 5 µL of the buffer was pipetted onto the NP-S oxide-sharpened silicon nitride tip and positioned on the AFM scanner head for imaging. AFM images were taken in a fluid tapping mode using Nanoscope III (Vecco, CA, USA) (Fig. 2).

### UV-vis spectroscopy

UV-vis spectroscopy of DNA, curcumin, metallo-curcumin, curcumin-conjugated DNA, and metallo-curcumin-conjugated DNA complexes was performed using Nano Drop 2000c (DE, USA) in the wavelength range of 200–800 nm. 2 µL of the sample was dispensed onto the pedestal before the absorbance measurement (Fig. 3(a)).

### FTIR measurement

FTIR spectra of DNA, curcumin-conjugated DNA, and metallo-curcumin-conjugated DNA complexes were recorded in the wavenumber range of 4000 to 700 cm^−1^ using a spectrometer with a resolution of 4 cm^−1^ (TENSOR 27; Detector: MIR_ATR (ZnSe); Bruker, MA, USA) (Fig. 3(b)) and Supplementary Table S3).

### TOF-SIMS measurement

Trace elemental and chemical fragment analysis on the surface of SDNA and SDNA-Cu-C complexes were performed using a reflection-based high-resolution TOF-SIMS-5 instrument (ION-TOF GmbH, Germany). Negative ion-based mass spectra and chemical mapping are acquired using a pulsed ion source with 25 keV and 1.3 pA primary Bi^3+^ cluster in the high-current bunched mode for 150 × 150 µm^2^ on the sample surface. (Fig. 4 and Supplementary Fig. S2).

### Drug release

Metallo-curcumin release rates were determined using a dialysis procedure. The metallo-curcumin-conjugated RDNA complexes were transferred into dialysis tubes that had been preconditioned with DI water for 15 min. Then, the metallo-curcumin-conjugated RDNA complexes in the dialysis tubes were dialyzed against 600 µL of 0.2 M phosphate-buffered saline (PBS) containing 0.5% Tween 20 in an incubator-shaker maintained at 37 °C and 200 RPM. At predetermined time intervals, 100 µL of the released medium was taken and analysed using fluorescence spectroscopy with an excitation wavelength of 420 nm (VICTOR Multi-label Plate Reader, Perkin Elmer 2030, Seoul, Korea) (Fig. 5).

### Bacterial growth kinetics

To assess the anti-bacterial activities of DNA, curcumin, curcumin-conjugated DNA, and metallo-curcumin-conjugated DNA complexes, pathological hospital bacterial strains—*Escherichia coli* (*E. coli*) for the Gram-negative and *Bacillus subtilis* (*B. subtilis*) for the Gram-positive category—were inoculated in LB medium. They were grown overnight in an orbital incubator-shaker at 37 °C. The culture was then diluted to an optical density (OD) of 0.1, and 100 µL of the culture was inoculated in the corresponding well of a 96-well plate. The OD absorbance of the culture medium was then recorded at 600 nm using an Epoch 2 Microplate spectrophotometer (BioTek Instruments, VT, USA) over a period of 20 h. The bacterial growth curve was found by optical density at 600 nm (OD_600_). The measurement was conducted three times (Fig. 6).

### Cell culture studies

(a) The prostate cancer cells (PC3, 22Rv1, TRAMP-C1, LNCaP, and DU145) were maintained in RPMI 1640 medium supplemented with 10% FBS and 1% Antibiotic-Antimycotic solution. The cells were cultured and incubated in a 5% CO_2_ environment at 37 °C in a CO_2_ incubator. Once the cells attained 85% confluency, they were detached from the flask with 0.25% trypsin-EDTA and centrifuged at 1500 RPM for 5 min. The cell pellet was then resuspended in a complete growth medium. The cells were counted using a hemocytometer and used for further cell culture experiments.

### Cell viability

Cell viability studies were carried out on PC3, 22Rv1, TRAMP-C1, LNCaP, and DU145 cell lines by the CellTitre 96 Aqueous One Solution Cell Proliferation Assay (MTS), a homogeneous, colorimetric method for determining the number of viable cells in proliferation, cytotoxicity, and chemosensitivity assays. The MTS was composed of solutions of a novel tetrazolium compound [3-(4,5-dimethyl-thiazol-2-yl)-5-(3-carboxymethoxyphenyl)-2-(4-sulfophenyl)-2H-tetrazolium, inner salt; MTS] and an electron coupling reagent (phenazine methosulfate; PMS). The MTS was bio-reduced by the cells into a formazan product that was soluble in tissue culture medium. The cells were seeded on a 96-well plate with a cell density of 5000 cells/cm^2^. Metallo-curcumin-conjugated DNA complexes with various metal ion concentrations (80 µM of curcumin with either 4 mM of Cu^2+^, 2 mM of Ni^2+^, or 1 mM of Zn^2+^) were prepared. The final concentrations of RDNA and SDNA were 500 nM and 0.1 wt%, respectively. Appropriate controls such as DMSO, 1 × TAE/Mg^2+^ buffer, and DI water were also tested in each case to compare the effect. After reaching 85% confluency, the media were removed and the cells were washed with DPBS. The metallo-curcumin-conjugated DNA complexes and fresh RPMI medium was added to a final volume of 100 µL in each well and incubated. The cells in medium alone (devoid of test samples) acted as a positive control and the cells treated with Triton X-100 acted as a negative control. The experiment was carried out for 24 and 48 h. After 24 and 48 h, 20 µL of MTS solution was added and the cells were incubated for 4 h to form the formazan product. The cell viability was then ascertained by measuring the OD of the culture at 490 nm using a BioRad iMark Microplate Reader (BioRad, CA, USA). This measurement was carried out in triplicate (Figs 7 and 8).

### Cell uptake

22Rv1 cells were seeded at a density of 5000 cells in a confocal dish attached to a cover slip and incubated for 24 h for cell attachment. After 24 h, the medium was removed and the cells were washed twice with PBS. Then, fresh RPMI medium was added to the cells along with the required concentration of metallo-curcumin-conjugated RDNA complexes. The cells were then incubated for 6 h at 37 °C in a 5% CO_2_ incubator. After incubation, the cells were washed thrice with PBS and fixed in 3.7% paraformaldehyde solution for 10 min, before being permeabilized with 0.2% Triton X-100 for 5 min. The cells were then further washed with PBS. Finally, the cells were imaged using fluorescence microscopy (EVOS FLoid Cell Imaging Station, ThermoFisher Scientific, Korea) (Fig. 9).

## Electronic supplementary material

SUPPLEMENTARY INFORMATION
