# Supplementary material for: Metallo-Curcumin-Conjugated DNA Complexes Induces Preferential Prostate Cancer Cells Cytotoxicity and Pause Growth of Bacterial Cells
Source: Sci Rep. 2018 Oct 8;8:14929. doi: 10.1038/s41598-018-33369-z (PMC6175843; doi:10.1038/s41598-018-33369-z)
Supplement: Supplementary file 1 — SUPPLEMENTARY INFORMATION [file 41598_2018_33369_MOESM1_ESM.pdf]

Supplementary Information

Metallo-Curcumin-Conjugated DNA Complexes Induces Preferential Prostate Cancer Cells Cytotoxicity and Pause Growth of Bacterial Cells

Srivithya Vellampatti[1‡], Gopalakrishnan Chandrasekaran[2‡], Sekhar Babu Mitta[1], Vinoth-Kumar Lakshmanan[2,3] & Sung Ha Park[1]

[1] Sungkyunkwan Advanced Institute of Nanotechnology (SAINT) and Department of Physics, Sungkyunkwan University, Suwon 16419, Korea.

[2] Department of Biomedical Sciences, Chonnam National University Medical School, Gwangju 61469, Korea.

[3] Department of Biomedical Engineering, Sri Shakthi Institute of Engineering and Technology, Coimbatore 641062, India

‡ These authors contributed equally to the paper.

Correspondence and requests for materials should be addressed to V.-K.L. (email: lvinothkumarbme@siet.ac.in) and S.H.P. (email: sunghapark@skku.edu).

Supplementary Fig. S1. DNA base sequences and pictorial representation of DNA rings.

Supplementary Fig. S2. Representative negative ion mode TOF-SIMS images.

Supplementary Table S1. RDNA base sequence information.

Supplementary Table S2. Sticky-end sequences for DNA rings.

Supplementary Table S3. FTIR absorption frequencies of SDNA and their spectral assignments.

Supplementary Fig. S1. DNA base sequences and pictorial representation of DNA rings. (a) A unit building block of RDNA containing two strands, Ring 1-1 and Ring 1-2. The complementary sticky-ends are indicated as s1, s1´ and s2, s2´. (b) Schematic diagram of an RDNA. The outer and inner diameters of the RDNA were 29 and 13 nm, respectively.

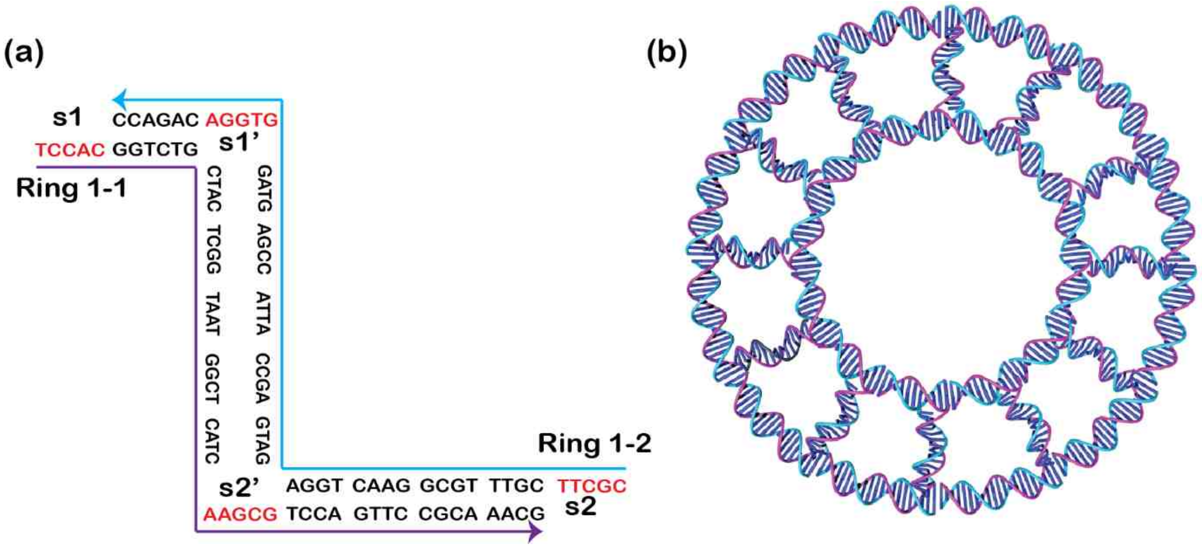

Supplementary Fig. S2. Representative negative ion mode TOF-SIMS images (scan size of 150 × 150 μm^2) showing the distribution of sugar, phosphate backbone, and nucleobases fragments in SDNA molecules and SDNA-Cu-C complexes.

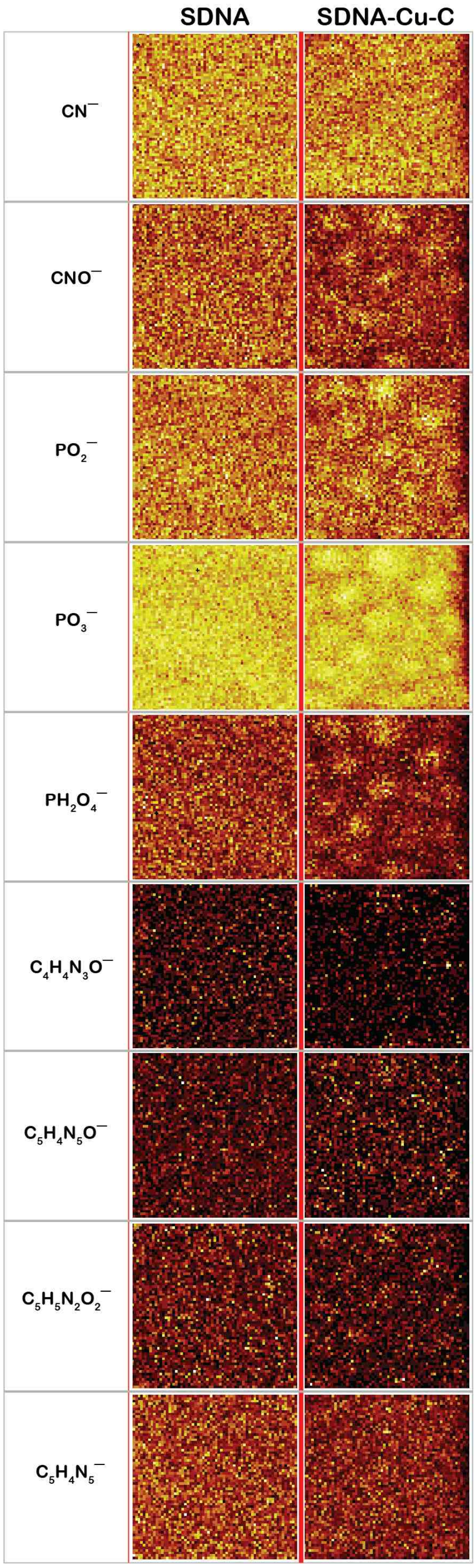

Supplementary Table S1. RDNA base sequence information: strand name, total number of nucleotides, and base sequence.

Strand Name	Total # of nucleotides	Sequence (5′ to 3′)
Ring 1-1	52	TCCA CGGT CTGC TACT CGGT AATG GCTC ATCA AGCG TCCA GTTC CGCA AACG
Ring 1-2	52	CGCT TCGT TTGC GGAA CTGG AGAT GAGC CATT ACCG AGTA GGTG GACA GACC

Supplementary Table S2. Sticky-end sequences for DNA rings (RDNA).

	5′ to 3′	3′to 5′	
**s1**	TCCAC	AGGTG	s1′
**s2**	CGCTT	GCGAA	s2′

Supplementary Table S3. FTIR absorption frequencies of SDNA and their spectral assignments.

DNA band position [Wavenumber (cm^{-1})]	Band assignment
780	Sugar vibrations
888	Deoxyribose ring vibrations
960	C-C and C-O stretching of the backbone
1018	C-O ribose
1058	C-O deoxyribose stretching
1083	PO_2^- symmetric stretch
1230	PO_2^- antisymmetric stretch
1371	Cytosine and Guanine
1486	Cytosine in-plane vibrations
1606	Adenine C7=N stretching
1648	Thymine (C2=O stretching)
3339	OH stretching
